# Wide Field-of-View Fluorescence Imaging with Optical-Quality Curved Microfluidic Chamber for Absolute Cell Counting

**DOI:** 10.3390/mi7070125

**Published:** 2016-07-20

**Authors:** Mohiuddin Khan Shourav, Kyunghoon Kim, Subin Kim, Jung Kyung Kim

**Affiliations:** 1 Department of Mechanical Engineering, Graduate School, Kookmin University, 77 Jeongneung-ro, Seongbuk-gu, Seoul 02707, Korea; khan@kookmin.ac.kr (M.K.S.); subinkim@kookmin.ac.kr (S.K.); 2 School of Mechanical Systems Engineering, Kookmin University, 77 Jeongneung-ro, Seongbuk-gu, Seoul 02707, Korea; 3 Department of Mechanical and Aerospace Engineering, North Carolina State University, Raleigh, NC 27695, USA; kkim15@ncsu.edu

**Keywords:** curved chamber, fluorescence imaging, aberration, field curvature, field of view, cell counting

## Abstract

Field curvature and other aberrations are encountered inevitably when designing a compact fluorescence imaging system with a simple lens. Although multiple lens elements can be used to correct most such aberrations, doing so increases system cost and complexity. Herein, we propose a wide field-of-view (FOV) fluorescence imaging method with an unconventional optical-quality curved sample chamber that corrects the field curvature caused by a simple lens. Our optics simulations and proof-of-concept experiments demonstrate that a curved substrate with lens-dependent curvature can reduce greatly the distortion in an image taken with a conventional planar detector. Following the validation study, we designed a curved sample chamber that can contain a known amount of sample volume and fabricated it at reasonable cost using plastic injection molding. At a magnification factor of approximately 0.6, the curved chamber provides a clear view of approximately 119 mm^2^, which is approximately two times larger than the aberration-free area of a planar chamber. Remarkably, a fluorescence image of microbeads in the curved chamber exhibits almost uniform intensity over the entire field even with a simple lens imaging system, whereas the distorted boundary region has much lower brightness than the central area in the planar chamber. The absolute count of white blood cells stained with a fluorescence dye was in good agreement with that obtained by a commercially available conventional microscopy system. Hence, a wide FOV imaging system with the proposed curved sample chamber would enable us to acquire an undistorted image of a large sample volume without requiring a time-consuming scanning process in point-of-care diagnostic applications.

## 1. Introduction

The term “aberration” is generally used to describe errors in optical images. Aberrations are caused by different focus positions on the image plane. Field curvature is one among the various types of monochromatic aberrations. For a flat detector surface, Petzval and field curvature must be corrected by balancing positive and negative power surfaces [1]. To overcome this problem, conventional microscope objectives are designed with multiple lenses. Although this usually corrects all aberrations, it is quite expensive to implement. The resulting system cost, size, and complexity limit accessibility of the device to well-funded laboratories with the required space and personnel trained in device operation and maintenance. Although unconventional solutions are becoming more popular for some applications, balancing performance and cost is challenging [2]. Smith et al. [3] proposed two common biomedical devices, a microscope and a spectrometer, as simple and inexpensive add-ons to a commercial cell phone camera. Although other researchers have previously demonstrated similar devices, such as mobile phone-based clinical microscope [4] that has both bright field and fluorescence imaging modes, the phone attachments proposed by Smith et al. are considerably smaller, simpler, and low in cost while still maintaining acceptable levels of performance. With increasing demand for fluorescence imaging in diagnostics, many imaging techniques have been developed. A cytometry platform [5] was designed and installed on a cell phone for blood cell analysis in field settings, enabling cost-effective and rapid measurement of the density of blood cells and hemoglobin concentration. Choi et al. [6] proposed and developed a fast-scanning method for fluorescence detection in three-dimensional (3D) cell culture by introducing a digital micromirror device (DMD), and Benjamin et al. proposed adaptive scanning optical microscopy (ASOM) [7], a unique wide-field imaging system designed with inexpensive techniques compared to conventional microscopy. However, the design of the latter is complex and might warrant careful handling.

Field curvature is a type of monochromatic aberration. Although all aberrations encountered with a conventional microscopic objective can be corrected using multiple lens elements, doing so is quite expensive. The advantages of a curved image plane have been described [8,9]. A curved imager provides a large degree of freedom in camera system design. It affords better resolution and brightness, in addition to reducing primary aberrations. Curved focal plane arrays (FPAs) have been proposed to remove spherical aberrations caused by spherical mirrors. Moreover, large field-of-view (FOV) cameras, spherical imagers, artificial retinas, and biomimetic optical sensors, equipped with curved FPAs have been proposed [10,11]. Jung et al. [12] recently made progress toward the realization of an eyeball camera. Realistic images can be collected by functional silicon devices. Despite the fact that these systems have advantages over a conventional planar detector, their curved detector with fixed curvature leads to discrepant variable zoom when using simple lenses.

A few lens-free approaches to point-of-care (POC) diagnosis have been proposed and advanced [13,14,15,16,17]. These may make the system more compact and easier to use for personnel. A giga-pixel fluorescent imaging system has been developed by modifying a commercially available scanner, which can analyze 2.2 mL of sample volume [18]. For lens-free imaging, samples are to be placed over the image sensor before image capture. Sample image does not have the optical aberration that may occur with lens imaging. However, the area of these images depends on the sensor size, and to place the sample over the sensor, a custom-fabricated chamber is required. Conventional imaging systems employ several lenses to eliminate primary aberration, because of which, designing systems for remote and on-site applications becomes expensive and complicated. These challenges can be overcome using a curved imaging surface to reduce the number of lenses in the imaging system and eliminate primary aberration and field curvature. However, sophisticated engineering skills are required to fabricate the designed system, and the curved imaging sensor might not be affordable to people from resource-confined areas. A curved refractive mirror was introduced as a substitute for correcting objective lens to compensate for field curvature or the Petzval effect [19]. The proposed system leads transmitted rays to a flat image plane and ensures uniform focus on the flat image plane for flat objects.

Herein, we describe a simple optical imaging system for large-field absolute counting of white blood cells (WBCs). An alternative approach is developed to correct field curvature in a flat image plane by replacing the conventional planar sample chamber with a curved one while using a single lens. The Petzval theory was revisited, and an optical simulation was conducted to determine the lens-specific curvature of the curved sample substrate that reduces field curvature in a compact single-lens fluorescence imaging system. The proposed method was validated further by experimental studies conducted using a custom-made curved sample substrate and an optical-quality curved sample chamber with fluorescent microbeads and white blood cells.

## 2. Materials and Methods

### 2.1. Theory

Petzval field curvature can be determined as below [20]:

(1) $$\frac{1}{r_{k}}=-n_{k}\sum_{i=1}^{k}\frac{K_{i}}{n_{i-1}n_{i}}$$

where *n_i_* is the refractive index of the *i*-th material, *r_i_* is the radius of curvature of the *i*-th material, and *K_i_* is the power of the *i*-th lens, defined as *K_i_* = (*n_i_* − *n*_*i*−1_)/*R_i_*, where *R_i_* is the radius of the *i*-th lens. The field curvature or Petzval radius of a single lens can be calculated as follows:

(2) $$\frac{1}{n_{0}r_{0}}-\frac{1}{n_{2}r_{2}}=\frac{K_{1}}{n_{0}n_{1}}+\frac{K_{2}}{n_{1}n_{2}}$$

where *r*_0_ and *r*_2_ are the radii of curvature of the object and the image surfaces, respectively. Because light is projected through a single lens, *n*_0_, *n*_1_, and *n*_2_ are the refractive indices after refraction in the three-media system. We found that *r*_0_ and *r*_2_ are symmetric to each other, and assuming the image surface is flat (*r*_2_ → ∞), Equation (2) reduces to:

(3) $$\frac{1}{n_{0}r_{0}}=\frac{K_{1}}{n_{0}n_{1}}+\frac{K_{2}}{n_{1}n_{2}}$$

### 2.2. Imaging Setup

We used Zemax (ZEMAX LLC, Kirkland, WA, USA), a ray-tracing simulation tool that helps model, analyze, and design optical systems, to optimize the design for minimal field curvature and reduced aberrations. Our model included one single biconvex lens, and the refractive material was N-BK7. At the start of the optimization process, the variables were sample object radius and fixed FOV to be imaged. The practicality of model assembly was kept by setting the object at a position beyond the focal length of the lens.

**Figure 1:**
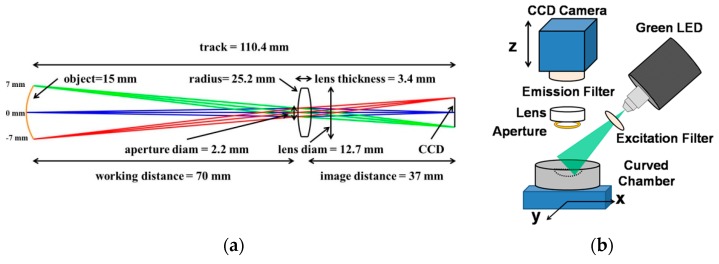
(**a**) Optical prescription for prototype curved-substrate imaging system, designed for a wavelength of 560 nm. A single commercial lens was used for imaging. All dimensions are in millimeters. (**b**) Schematic of experimental setup.

In our optics setup, a fluorescence imaging platform and fluorescent micro-objects located within a large-area curved substrate were designed as shown in Figure 1a. The working distance increases because of the large FOV target, which ensures that sample handling is comfortable.

A commercial CCD camera sensor (DMK 51BUC02, ImagingSource, Bremen, Germany) with a resolution of 1600 × 1200 pixels and a pixel size of 4.4 µm was used. We used a biconvex lens (LB1014, Thorlabs, Newton, NJ, USA), the focal length and refractive index of which were 25 mm and 1.517, respectively. The lens, an emission filter (XF3083, Omega Optical, Brattleboro, VT, USA) and the camera were aligned with a C-mount extension tube (25 mm length, #58-736, Edmund Optics, Barrington, NJ, USA) and a C-mount lens mount (12.5 mm diameter, #55-246, Edmund Optics) was then fixed with a dovetail stage (22 mm travel, #03-682, Edmund Optics). These integrated parts of optics enable the system to move along the *z*-axis to adjust the focus. An *x-y* positioning stage was attached with the integrated part to keep a rigid distance between the lens and the sample chamber as illustrated in Figure 1b. The light source was fixed separately, as shown in the schematic. This structure makes the system robust and not too sensitive.

### 2.3. Fabrication of Curved Sample Chamber

**Figure 2:**
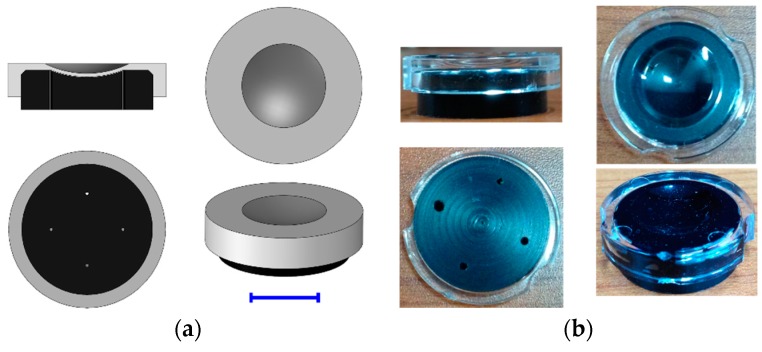
(**a**) 3D model of curved sample chamber composed of an upper window (gray color) and a lower substrate (black color). A sample solution is introduced into the gap of 100-μm thickness between those two separable parts through one of the holes in the lower substrate. The other three holes are air vents. (Scale bar = 10 mm). (**b**) Curved sample chamber fabricated by plastic injection molding technique. The upper window is optically clear for image acquisition and the lower substrate is colored black to minimize light reflection at the surface.

Images produced by the two optical systems were simulated in an accurate model by using Zemax software and considering relative illumination, geometric aberration, and point spread functions (PSFs) at various heights on the sample substrate. Following the proof-of-concept simulation with the curved sample substrate, we designed a curved sample chamber consisting of two parts, an upper window and a lower substrate. Both parts have spherical dome shapes with lens-dependent curvatures and can be snapped together manually with a gap of 50–500 μm to hold a fixed sample volume. A sample solution can be loaded into the chamber through a hole made in the lower substrate. The volume of the sample filled in the chamber is proportional to the gap thickness between the window and the substrate. Figure 2a shows a 3D model of the curved sample chamber. To fabricate the disposable optical-quality curved chambers, we used a transparent acrylic material for the upper window which was prepared using a plastic injection molding technique. The lower substrate was colored black to reduce a reflective light at the surface. The cost for making a mold for the sample chamber is about 4000 USD and we have produced 200 pieces for our study. The cost per piece is reduced exponentially with the number of pieces in a batch produced by injection molding process. Large FOV fluorescence imaging is enabled by a simple lens (~20 USD) and a curved sample chamber without using a sample scanning mechanism, which reduces the system cost greatly. Figure 2b shows various views of the fabricated curved sample chamber. For comparing the fluorescent particle images taken in the curved chamber with those taken in the planar chamber, we used the central part of a glass bottom culture dish (P35G-0-14-C, MatTek, Ashland, MA, USA) covered with a circular coverslip as a planar sample chamber.

**Figure 3:**
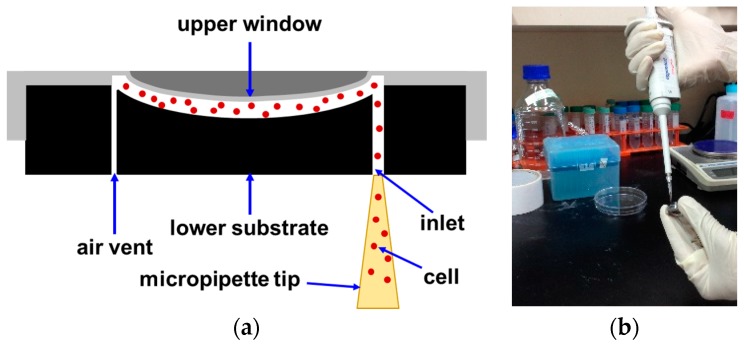
(**a**) A schematic of the cross-sectional view of the curved sample chamber and suspended cells in the gap between upper window and lower substrate; and (**b**) loading of cell suspension with a micropipette is facilitated by air vent holes made through the lower substrate as shown in (**a**).

A schematic of the cross-sectional view of the sample chamber with the distribution of the cells is depicted in Figure 3a. Cells are distributed across the gap rather than deposited at the curved bottom part. A large field-of-view image of the cells is taken immediately after loading the sample into the chamber, and thus even those cells with a high density are not deposited on the bottom during image acquisition. As the field of view of our imaging system is large and the depth of focus is greater than the gap thickness, cells are imaged sharply regardless of their positions inside the chamber. It is quite easy to load a cell suspension into the sample chamber by a micropipette as shown in Figure 3b, as there are air vents in the lower substrate. No leakage is observed through the air vents.

Our curved chamber was designed to be used as a disposable one to prevent infection. If there is no concern about infection, it can be reused after separating the upper window and the lower substrate and cleaning each part with a distilled water or a diluted ethanol solution. It does not take long or require a tedious process for cleaning as, cells are not adhered to the bottom substrate.

### 2.4. Sample Preparation

A small amount of whole blood was donated by members of our laboratory. We used 1× RBC (red blood cell) lysis buffer by diluting the original 10× RBC lysis buffer to 1× working concentration with deionized water (DW). A 10:1 ratio was maintained to form 1× RBC lysis buffer. Before using, we keep the buffer at room temperature for 10 min. Then, we added 2 mL of 1× RBC lysis buffer to each e-tube containing up to 100 μL of whole blood. For mixing, we gently vortexed each tube immediately after adding the lysed solution. We maintained the vortexed tubes at room temperature for 10–15 min. We centrifuged the sample at 500 G for 5 min. Then, we pulled out carefully the upper part of the sample without disturbing the pellet. Then, we washed the sample by adding 1× PBS (phosphate buffered saline) + 2 mM EDTA (ethylenediaminetetraacetic acid) + 0.2% BSA (bovine serum albumin) and suspending the cell pellet with 4 mL PBS. Then, we centrifuged the samples for 5 min and collected the supernatant.

We prepared 8 different samples with different WBC concentrations. After extracting WBCs from whole blood, we mixed it with different quantities of PBS buffer. The prepared samples had WBC concentrations of 100%, and 10%–70% with an interval of 10%. The WBC samples were then stained with a fluorescence dye. We added 20 µL of 1 mM SYTO82 orange fluorescent nucleic acid stain (Thermo Fisher Scientific, Waltham, MA, USA) to 200 µL of each sample solution.

### 2.5. Image Acquisition and Cell Counting

We imaged WBCs with conventional microscopy using a 4× magnification lens with an exposure time of 30 ms in a handmade slide glass chamber, which covers a FOV of 2.2 mm × 1.65 mm, and the volume enclosed by this area is 0.8 µL. Chamber height is 220 µm, and the total area is 180 mm^2^ (15 mm × 12 mm), which makes the total volume 40 µL. ImageJ software was used to binarize the image to count WBC manually. The raw images were then processed using a FFT bandpass filter, which subtracts the background from the image. Images of large structures over 40 pixels and small debris less than 3 pixels were removed. With the single-lens imaging system, we imaged the same chamber and our designed curved chamber with an exposure time of 30 ms to directly compare WBC counts. The size of the image is 10.66 mm × 8 mm and the enclosed sample volume is 18.76 µL for flat chamber. Likewise a curved chamber was also imaged in the single lens imaging system with the same area covered but the volume was 8.51 µL. The height of the curved chamber between the cover and the base was 100 µm and chamber diameter was 12.7 mm, which means the chamber could hold 12.67 µL of sample. This is around three times lower than the volume of the planar chamber. The magnification factor of the single-lens imaging system was 0.66.

## 3. Results and Discussion

### 3.1. Simulation Study

Zemax software was used to model the imaging system to determine the aperture radius, lens-to-camera distance, and optical resolution, as well as to simulate the image on the image plane. However, the aperture radius was optimized independently.

**Figure 4:**
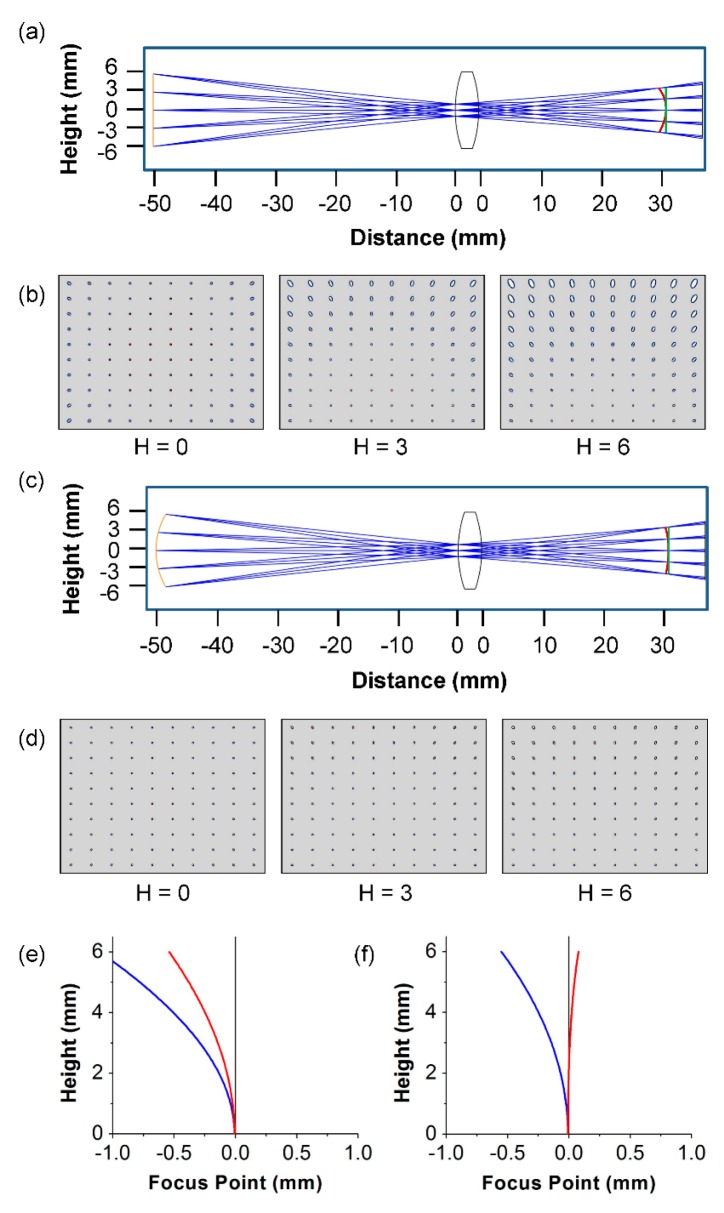
Ray tracing simulation of astigmatism field curvature. Ray tracing of a simple biconvex lens for: (**a**) flat substrate; and (**c**) curved substrate. The grayscale images in (**b**,**d**) were acquired using the flat and the curved substrates, where the image plane was kept flat. The images in (**b**,**d**) represent 0, 3, and 6 mm sample substrate height. The field curvature graphs for the flat and the curved substrates are shown in (**e**,**f**), respectively, where the blue and red lines denote tangential and sagittal surfaces.

The model included one single biconvex lens (LB1450, Thorlabs) made of refractive material N-BK7, an aperture, object at a fixed distance to achieve a certain FOV, and a flat image plane. The lens diameter was 12.7 mm, thickness 3.9 mm, focal length 20 mm, and radius of curvature 19.9 mm, and it was placed 50 mm from the sample substrate. An aperture stop of 2 mm was mounted toward the front surface of the lens. The optics simulation began with the flat substrate and a preset FOV, as shown in Figure 4a. The software was initially allowed to optimize the image by viewing a dot grid model. Then, the calculated substrate curvature was simulated, as shown in Figure 4c, and the dot grid was analyzed. The dot grid was simulated at three different heights on the flat and calculated curved substrates, as shown in Figure 4b,d, respectively. Optimally focused images were obtained when the radius of curvature of the substrate was kept within the sagittal value of the lens, as shown in Figure 4b. Figure 4e,f shows a quantitative analysis consisting of field curvature graphs for flat and curved substrates, respectively. The red and blue lines represent the sagittal and tangential surfaces, respectively. The on-axis position (H = 0 mm) in this graph is considered the center of the 12 mm substrate. The focus points are deviated from the image plane with increasing height. However, in Figure 4f, the sagittal surface becomes more flat and aligned with the image plane. Thus, the curved substrate can focus the rays to a point in the flat plane.

The sagittal surface was calculated by first determining mathematically the Petzval surface, a natural characteristic that exists in every optical system. The ratio of the distance between the Petzval surface and the tangential and sagittal surfaces is 3:1. The sagittal surface can thus be calculated as follows:

(4) $$\frac{1}{R_{s}}=\frac{1}{R_{p}}-\frac{1}{f}=\frac{1+n}{nf}$$

where *R_s_* and *R_p_* are the radii of the sagittal and the Petzval surface curvatures, respectively; *n* is the refractive index of the medium; and *f* is the focal length. From Equations (3) and (4), for the lens used in this study, the Petzval sagittal radii are −29.19 mm and 12 mm, respectively.

**Figure 5:**
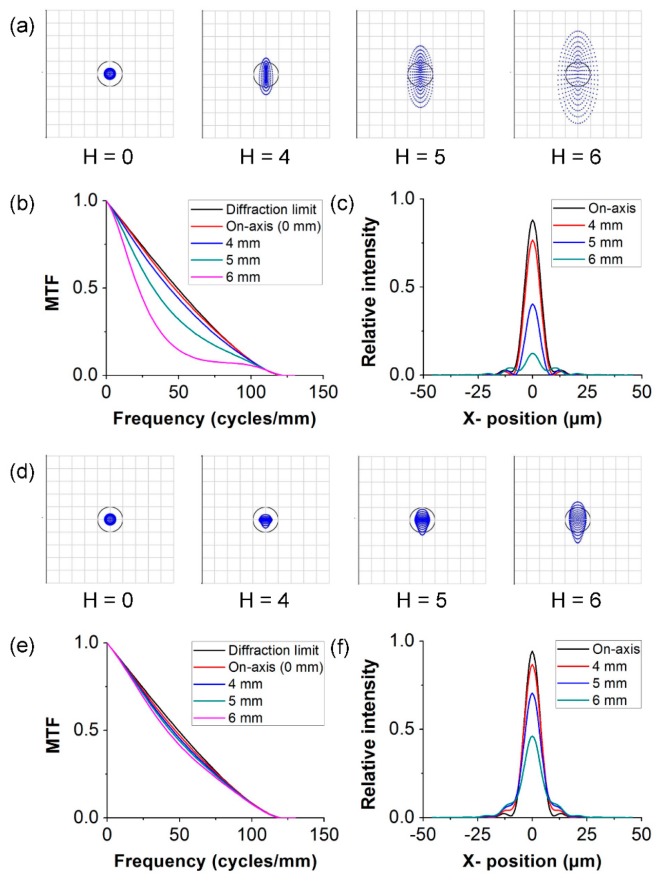
Analytical characterization of simple imaging system for flat and curved substrates. The spot size diagrams include a circle showing the Airy disk (9.87 μm in radius) for the flat and the curved substrates in (**a**,**d**), respectively. (**b**,**e**) MTF and (**c**,**f**) PSF of flat and curved substrates, respectively. In the MTFs, the black line shows the diffraction limit, and the red and pink lines represent the on- and off-axis image fields, respectively.

The designed optical system was simulated by a ray-tracing numerical model using Zemax software. Zemax helps confirm the results of the analytical model and evaluate optical performance over the FOV. The spot diagram, modulation transfer function (MTF), and point spread function (PSF) of the curved substrate show considerably better agreement (Figure 5) than those of the flat substrate. The radius of the Airy disk was 9.87 µm for both the flat and the curved substrates in the optical system. The RMS radius of the spot diagram in Figure 5a diverges with increasing field height for the flat substrate, but it remains in the Airy disk radius for the curved substrate, as shown in Figure 5d. Numerical modeling results obtained across the FOV are shown in Figure 5b,e, where the optimal FOV was used to calculate the MTF. The MTF of this system is plotted for a field point on the optical axis and another point at the edge of the FOV. The field point on the optical axis shows a nearly diffraction-limited response at a spatial frequency of about 120 cycles/mm for the curved substrate in comparison with that for the flat substrate [21].

The PSFs on the image plane at 560 nm are shown in Figure 5c,f. The advantage of a curved substrate is clear on the image plane. This PSF represents light intensity over the entire FOV on the imaging surface. The on-axis image intensities are similar to each other but vary with the field height. The off-axis PSF of the curved substrate exhibits a higher peak than that of the flat substrate, and it is more uniform with respect to the wavelength and image height, thus resulting in better image formation.

### 3.2. Validation Study

**Figure 6:**
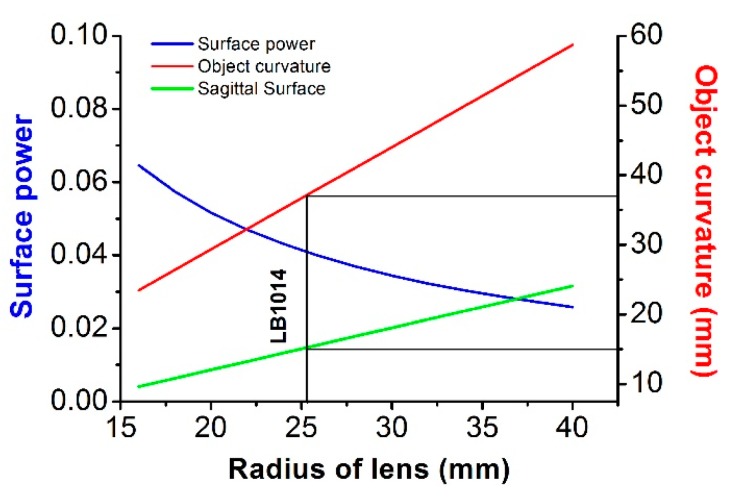
Change in surface power and object curvature with change in curvature radius of lens. These radii of the lens consider both sides of biconvex lens, and have the same focal length as the radius.

The curvature radii of objects used in this experiment were calculated for different lenses considering their field curvature radii. Curved objects were fabricated as described in the Materials and Method Section. Figure 6 shows the change in object radii with a change in lens radius. The Petzval radii of the biconvex and the plano-convex lenses used in our experiment were calculated.

We determined the object curvature using Equation (2), while the image surface was kept infinity. Finally, we calculated the power of the lens surface as follows:

(5) $$\frac{n_{1}-n_{0}}{R_{1}}=K_{1}$$

By using the above formulas, we found the Petzval radius and the surface power of the lens. Here we used a commercial lens LB1014 for designing the chamber. Table 1 below lists the specifications of lenses with different curvature and because the FOV is fixed, the volume varies.

Figure 6 shows plots for a certain FOV, and it can be seen that upon increasing the FOV, system length increases. Thus, choosing a short focal length lens can reduce the size of the imaging system.

**Figure 7:**
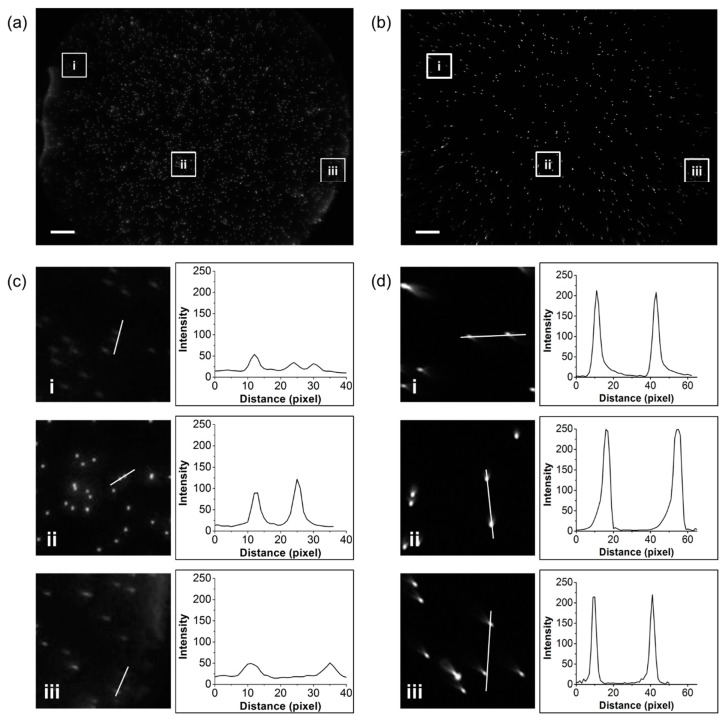
Imaging performance of single-lens fluorescence microscopy with curved sample chamber. Fluorescence images taken for beads of diameter 10 μm for: (**a**) planar; and (**b**) curved sample chambers. The fluorescent microbeads are distributed uniformly over the entire FOV. The intensity profiles of the bead images are shown in (**c**,**d**). As observed for the curved substrate, a wider area of the bead image is more focused in (**b**), and the aberration is lower than that in (**a**). (Scale bar = 1 mm).

A validation study was conducted with the planar and curved sample chambers, in which a fluorescent microbead suspension was loaded. The imaging setup is discussed in the Methods Section, and a schematic diagram is shown in Figure 1b. As shown in Figure 7a,b, the fluorescent microbeads were distributed uniformly over the entire FOV. The brightest circular region in the center of the image shown in Figure 7b is attributed to the fluorescent microbeads aggregated at the inlet hole. It can be removed by changing the position of the hole in the lower substrate. In our curved sample chamber, the well-focused area is approximately 119 mm^2^, which is twice as large as the approximately 64 mm^2^ aberration-free area of the planar chamber. Furthermore, the image in the curved chamber also exhibits almost uniform brightness over the FOV (Figure 7d), while, in the case of the planar chamber, the distorted portion at the edge has much lower intensity than the central region (Figure 7c). The fluorescent microbeads positioned at the edge part of the curved chamber exhibit a small distortion, which is attributed to other type of optical aberration such as coma. As mentioned earlier, our curved sample chamber with a single-lens imaging system can reduce the field curvature only and enlarge the focused area of the image.

### 3.3. Absolute Counting of WBC

**Figure 8:**
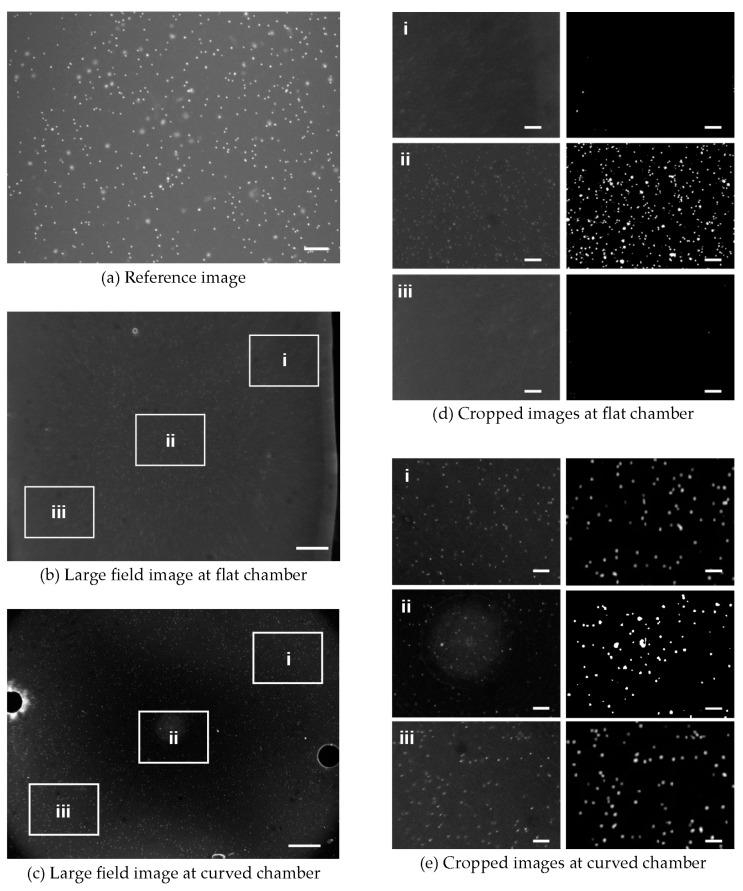
Fluorescence images of stained white blood cells in small and large field imaging. (**a**) Microscopic image of flat chamber. The inset white boxes in (**b**,**c**) are of the same size in the microscopy images of the flat and curved chambers. (**d**,**e**) Cropped areas of the flat and curved chamber images, where (ii) denotes the center part of an image. All cropped images are of the same size. The cell counts in the center (ii) were similar for both chambers. The bars in (**b**,**c**) represent a scale of 1 mm, while those in the other figures represent a scale of 200 µm.

We investigated the feasibility of our curved chamber platform by analyzing SYTO82 stained WBCs at different locations within the chambers. The image shown in Figure 8a has an area of 2.2 mm × 1.65 mm of flat chamber achieved using 4× magnification and Figure 8b,c show images of the flat and the curved chambers taken over a large field with an area of 10.66 mm × 8 mm. Portions corresponding to the 4× magnification area were digitally cropped from the whole images of the flat chambers as shown in Figure 8d. For comparison purpose, the same regions of the sample were also cropped from the curved chamber as shown in Figure 8e. For the flat chamber, the area cropped from the edge part shows the presence of few cells, which decreases the average number of cells in total. However, the cropped image of the curved chamber shows uniform number of cells throughout, and the average cell count deviates negligibly across the image. WBC detection at the edges of both the flat and the curved chambers showed a significant difference. The cell count at the edge of the flat chamber was very low, as shown in Figure 8d (i and iii), and, as expected from our validation study, the cell count at the edge of the curved chamber was higher (Figure 8e (i and iii)), which attributes to the uniform fluorescence intensity in the curved chamber image. As some monochromatic aberrations still exist in our single-lens imaging system, there are limited applications such as cell counting. However, our approach serves wide FOV absolute cell counting by reducing the field curvature effect.

**Figure 9:**
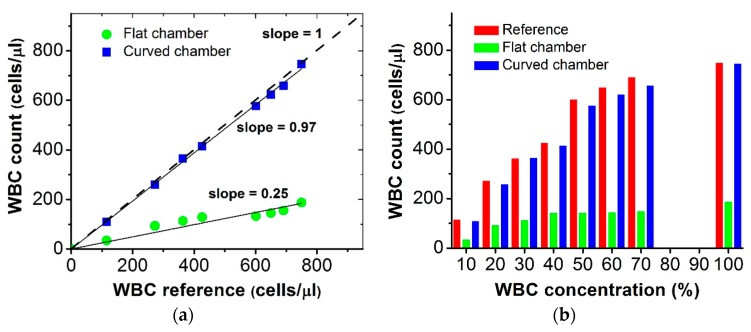
(**a**) WBC count obtained using planar chamber is remarkably different from the reference cell count obtained with conventional microscopy; and (**b**) WBC concentrations of 10%–70% with an interval of 10% and 100% were compared for absolute counting obtained by microscopic, flat, and curved chamber imaging. The count obtained with the curved chamber is close to that obtained with conventional microscopy. However, the corresponding result with the flat chamber differs considerably for large fields.

The main purpose of this research is to determine the absolute count of WBCs, defined as the total number of WBCs in a microliter of blood, in the designed chamber. By considering the microscopy result as the reference, we analyzed the flat and curved chamber images for WBC counts obtained from samples with eight different dilution levels. As shown in Figure 9a, a very good agreement is found between the counts using the reference microscopic images and the curved chamber images. However, the results obtained with the large flat chamber and those obtained with the curved chamber are considerably different from those obtained using conventional microscopy. The slope between the reference and the flat chamber is 0.25, while that between the reference and the curved chamber is 0.97.

In Figure 9b, the bar chart shows the average cell counts for the microscopic, flat and curved chamber imaging. Among the eight samples, the maximum deviation for the WBC counts from whole images obtained using the single-lens imaging system is found to be 5.12% at WBC concentration of 70%.

**Figure 10:**
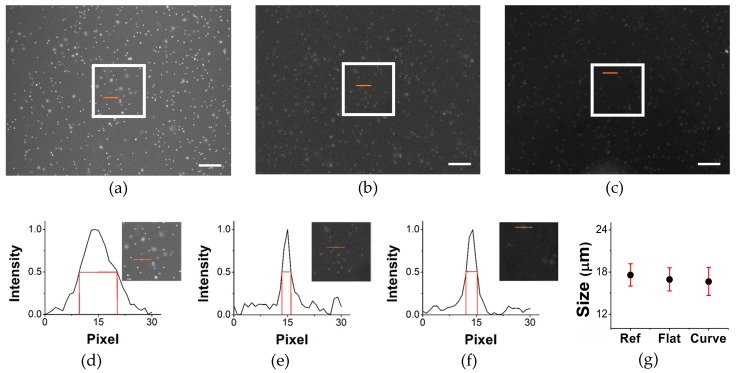
(**a**–**c**) The reference, flat, and curved chamber images with 500 µm^2^ white boxes inside, respectively (scale bar = 0.5 mm); (**d**–**f**) the normalized intensity profiles of a fluorescently-labeled WBC in those three images; and (**g**) the size of the fluorescent WBCs measured with microscopy, and flat and curved imaging, represented by Ref, Flat, and Curved on the *x*-axis of the graph, respectively. The range of WBC size is 16–20 µm. The normalized intensity profile of the fluorescence shows the size of the SYTO-stained WBC in pixels, and the inset images are cropped from the center part of the processed image.

We measured WBC size in two imaging methods to optimize our count results. This validation study confirms the same particle counting inside the chamber. Same size of the microscopic image is cropped at the center part from the large field of view image. To justify the WBC size obtained with both imaging systems, we measured the number of pixels occupied by the fluorescent dye. In Figure 10, the bottom panel shows the normalized intensity profiles of fluorescently-labeled WBCs. However, fluorescence covers an area greater than the particle size owing to the emission of light. We analyzed the full width at half maximum (FWHM) to measure WBC size, which is shown with red line in Figure 10d–f. The average number of pixels in the microscopic image is 11, and because 1376 pixels span a length of 2.2 mm, the size of an individual WBC cell is 17.58 µm. In the single-lens images, each fluorescence signal covers 2.5 pixels, and 1600 pixels span 10.66 mm; hence, the size of an individual WBC cell is 16.65 µm. It should be noted that those three images have different noise levels and signal-to-noise ratios as we compared our single lens imaging system with a conventional microscopy. In addition, for the single lens imaging system, difference in signal-to-noise ratio (SNR) can be seen because of different material properties of the sample chambers.

### 3.4. Advantages of Large FOV Imaging with Curved Sample Chamber

Using an eight-megapixel cell phone camera, Zhu et al. [22] achieved a FOV of approximately 81 mm^2^ in the central region of an approximately 162 mm^2^ image with a magnification factor (MF) of 0.3. The curved substrate enables us to enlarge the FOV by approximately 50% at an even higher MF of approximately 0.6. The FOV can be widened further if we use a lens with a longer focal length, while maintaining the MF within a reasonable range. Owing to this large FOV, an imaging system incorporating a curved sample chamber can image a large sample volume without a time-consuming scanning process, which is particularly important for rapid screening of samples such as blood, water, and urine. We demonstrated that an optical-quality curved sample chamber can be fabricated by the plastic injection molding process, which is beneficial from the viewpoint of lowering the cost of mass-producing disposable curved sample chambers. For laboratory-scale research, 3D printing with a transparent polymer resin can be employed as an alternative method for fabricating the curved sample chamber [23].

Rogers’ group developed an electronic eye, in which the image sensor was designed by considering the Petzval surface. Although other groups have reported the advantage of a curved focal image plane, meticulous engineering is required for fabrication. As we aimed to make a simple imaging system, we used a curved chamber instead of a curved image sensor. Despite the substantial reduction in field curvature, other aberrations still exist in the fluorescence image taken in the curved chamber because we use a single-lens imaging system and do not use any lens- or image-processing scheme to enhance image quality. The Petzval surface works only when the imaging system is corrected for astigmatism [24]. Note that the constant distance between the sagittal and the tangential surfaces in Figure 4e,f is attributed to the slightly deformed object (astigmatism) at the periphery of the image shown in Figure 7d. Nevertheless, those regions with aberrations are available for particle counting in the fluorescence imaging mode.

The aberration field curvature is related to astigmatism, but it might exist in a system that does not suffer from astigmatism. In the case of field curvature, an object is imaged on a curved surface rather than on a plane. The image is not blurred by this aberration, but it is simply projected onto a curved surface. This cannot be focused on with a flat sensor for flat object imaging. Our experimental and computational analyses demonstrate that the image can be focused on to a greater extent so no particles in the sample are lost. This is useful from the viewpoint of counting particles or cells in a large sample volume.

A traditional microscope can achieve high resolution over the entire image field. However, in terms of FOV, it can only capture a small area. Potsaid et al. proposed an adaptive scanning optical microscope (ASOM) by designing a unique wide field imaging system using an inexpensive technique compared to conventional microscopy. They introduced a microelectromechanical system (MEMS)-based deformable mirror with complex design of lens array. A high-throughput imaging system without a lens was proposed by Arpali et al. [25] for imaging large sample volumes. A special microfluidic chamber was designed in this case, and they system achieved good resolution over the image field. As the chamber was placed just over the CCD image sensor, it did not show the aberrations in the imaging system caused by a regular lens. However, a large CCD sensor was used, and it required sophisticated expertise in optics design. Previously, we developed a dual-mode high-throughput microscopy system to scan a wide field of asbestos slide sample [26]. Two translational stages of this system move along the *x*- and *y*-directions to acquire a series of small images over a large area of the sample slide. One of the drawbacks of this system is a limited FOV.

The design of a simple imaging system with a curved chamber has not received much attention, partly because calculation of the curvature varies with lens specifications, and there is no practical, low-cost technique for large-scale diagnostic imaging. Large FOV images have a number of advantages, and we can reduce the standard deviation of cell count by using large sample volume, which would allow us to count all cells in a single image. Different image areas from a single sample can have different amount of cells, which can be confusing for sample analysis. The proposed curved sample chamber and practical diagnostic imaging tool can be applied to point-of-care diagnosis such as absolute cell counting and detection of parasites in resource-constrained areas.

High precision in cell counting is vital for reliable diagnosis. A statistical term, coefficient of variation (CV), is used for the measurement of precision and calculated as

(6) $$CV(\%)=\frac{100}{\sqrt{n}}$$

where, *n* represents the number of cells counted in a sample [27]. In our study, the sample of lowest concentration contains 92 cells per 0.8 µL and 747 cells per 6.8 µL for 4× and 0.66× magnified images, respectively, and CVs are given as 10.4 and 3.65, respectively. For the sample of highest concentration at both magnifications, counts are 600 cells per 0.8 µL and 5077 cells per 6.8 µL, where CVs decrease to 4.08 and 1.40, respectively. Higher number of cells counted in a sample gives less variation and higher precision in absolute cell counting. Thus, a single image containing a large sample volume can decrease CV greatly, which is a strong advantage of large FOV imaging with our curved sample chamber.

## 4. Conclusions

We proposed an optical-quality curved sample chamber to reduce the field curvature, which is inherently unavoidable in optical imaging of a flat substrate with a simple lens system. Ray tracing simulations and experiments demonstrated that the optical distortion at the planar camera sensor could be corrected by using a curved sample chamber with a lens-dependent curvature. An application to absolute cell counting for the fluorescently-labeled white blood cells confirmed that a large field-of-view imaging with our curved sample chamber can reduce the variation in counting cells while increasing the precision of measurement. The present study supports the use of an unconventional curved sample chamber for designing a compact and cost-effective single-lens fluorescence imaging system.

## Figures and Tables

**Table 1:** Specification of imaging system for simulation.

Lens	Inner Surface of Sphere			Outer Surface of Sphere		
	Radius	Height	Volume	Radius	Height	Volume
	(mm)	(mm)	(mm^3^)	(mm)	(mm)	(mm^3^)
LB1014	15.06	1.25	71.88	15.56	1.75	144.09
LB1258	18.08	1.03	59.114	18.58	1.53	132.89
LB1378	24.12	0.8	47.96	24.62	1.3	128.41
LB1844	30.135	0.605	34.42	30.635	1.105	116.10
